# Breathing cessation events that compose the apnea–hypopnea index are distinctively associated with the adverse outcomes in Alzheimer’s disease

**DOI:** 10.1186/s13195-023-01266-x

**Published:** 2023-07-14

**Authors:** Adriano D. S. Targa, Iván D. Benítez, Anna Moncusí-Moix, Farida Dakterzada, Olga Minguez, Rafaela Vaca, Mireia Dalmases, Manuel Sanchez-de-la-Torre, Ferran Barbé, Gerard Piñol-Ripoll

**Affiliations:** 1 Translational Research in Respiratory Medicine (TRRM), Hospital Universitari Arnau de Vilanova-Santa Maria, Biomedical Research Institute of Lleida (IRBLleida), Lleida, Spain; 2 Centro de Investigación Biomédica en Red de Enfermedades Respiratorias (CIBERES), Instituto de Salud Carlos III, Madrid, Spain; 3 Cognitive Disorders Unit, Clinical Neuroscience Research, Hospital Universitari Santa Maria, Biomedical Research Institute of Lleida (IRBLleida), Lleida, Spain; 4 Precision Medicine in Chronic Diseases, Hospital Universitari Arnau de Vilanova-Santa Maria, Biomedical Research Institute of Lleida (IRBLleida), Lleida, Spain

**Keywords:** Alzheimer’s disease, Obstructive sleep apnea, Apnea–hypopnea index, Hypopneas, Cognitive decline

## Abstract

**Background:**

Previous studies challenge the impact of obstructive sleep apnea (OSA) once patients are diagnosed with Alzheimer’s disease (AD). Nevertheless, OSA recognizably disrupts sleep, and relevant associations between sleep, AD pathological markers, and cognition have been demonstrated. We aimed to further explore this, evaluating the associations between each breathing cessation event that compose the apnea–hypopnea index (AHI) and the sleep structure to finally investigate whether this was related to increased levels of AD markers and higher cognitive decline.

**Methods:**

Observational, prospective study, including consecutive patients diagnosed with mild-moderate AD. The participants were submitted to overnight polysomnography followed by a cerebrospinal fluid collection for AD pathological markers levels determination. Neuropsychological assessment was performed at baseline and after 12 months of follow-up.

**Results:**

The cohort was composed of 116 patients (55.2% females) with a median [p25;p75] age of 76.0 [72.0;80.0] years and an AHI of 25.9 [15.1;48.5], which was mainly defined by the presence of hypopneas and obstructive apneas. These were distinctively associated with the sleep structure, with obstructive apneas being related to arousals and sleep lightening and hypopneas being related to an increased number of arousals only. Despite having a lower frequency, mixed and central apneas also presented associations with the sleep structure, particularly increasing the time spent in the lighter sleep stages. In relation to AD pathological markers, obstructive and mixed apneas were related to an augment in neurofilament light levels while hypopneas were associated with a higher phosphorylated-tau/amyloid-beta protein ratio. Hypopneas were the most important event for an increased cognitive decline at the 12-month follow-up.

**Conclusions:**

Our findings highlight the importance of a patient-centered approach, with a comprehensive and detailed analysis of the AHI to effectively predict the different outcomes and tailor the appropriate therapeutic strategies.

**Supplementary Information:**

The online version contains supplementary material available at 10.1186/s13195-023-01266-x.

## Background

Alzheimer’s disease (AD) is the most prevalent neurodegenerative disorder in the world, currently affecting more than 55 million people [1]. Female sex and the presence of the apolipoprotein E (APOE) ε4 allele are two non-modifiable features associated with an increased risk of developing the disease [2]. Given that aging is also a relevant non-modifiable risk factor, estimations are that the prevalence of AD will increase in the next decades, especially considering the growing number of older adults in the nowadays society [1, 3]. Aiming to prevent this and/or reduce the progression of the disease, considerable attention has been dedicated to the modifiable factors associated with increased risk of AD such as hypertension [4], diabetes [5], obesity [6], and obstructive sleep apnea (OSA) [7], among others.

The presence of OSA among cognitively healthy subjects is associated with atypical levels of AD pathological markers [8–10], increased cognitive decline [11, 12], and a higher risk of mild cognitive impairment/dementia [13–16]. Nevertheless, previous studies suggest a different scenario once patients are diagnosed with AD. Accordingly, brain amyloid burden or cerebrospinal fluid (CSF) levels of phosphorylated-tau (P-tau) and total-tau (T-tau) presented similar alterations among AD patients with and without OSA after 2.52 ± 0.51 years of follow-up [17]. In addition, the presence of OSA was not associated with an increase in the magnitude of cognitive decline among patients with mild-moderate AD after three years of follow-up [18]. Yet, OSA recognizably disrupts the sleep structure, and previous research reported relevant associations between sleep lightening, atypical levels of AD pathological markers, and increased cognitive decline [19, 20].

OSA is currently diagnosed based on the apnea–hypopnea index (AHI), a metric composed of distinct events which are not properly evaluated for their individual contribution in terms of adverse outcomes. To address this and improve the understanding concerning the impact of OSA among patients diagnosed with AD, we first performed a comprehensive characterization of the breathing cessation events that compose the AHI among our cohort of consecutive patients with mild-moderate AD. In the sequence, we evaluated the impact of each of these events on sleep structure. Finally, we investigated whether the events associated with increased disruption of the sleep structure would be the ones related to atypical levels of AD pathological markers and higher cognitive decline.

## Methods

### Study population

This is an ancillary study of a prospective trial designed to evaluate the influence of OSA on the cognitive decline of AD patients after one year of follow-up (NCT02814045). The recruitment started in 2015 and finished in 2019. We included acetylcholinesterase inhibitor-naïve individuals aged over 60 years who were diagnosed with AD according to the National Institute on Aging and Alzheimer’s Association (NIA-AA) clinical criteria [21] and presented a Mini-Mental State Examination (MMSE) score ≥ 20. We excluded individuals presenting at least one of the following characteristics: (i) presence of visual and/or communication difficulties that could influence the compliance with the procedures of the study; (ii) presence of a previously diagnosed sleep disturbance; (iii) comorbidities such as cancer, severe renal or hepatic insufficiency, severe cardiac or respiratory failure; (iv) excessive alcohol intake (> 280 g/week); (v) MRI evidence of hydrocephalus, stroke, space-occupying lesion, or any clinically relevant disease of the central nervous system other than AD; (vi) presence of mental disorders according to DSM-V-TR™ criteria; (vii) use of medications under investigation or use of beta-blockers, antidepressants, neuroleptics, or hypnotics during the 15 days previous to polysomnography (PSG); (viii) presence of untreated (or treated for less than 3 months prior to the screening visit) vitamin B12 or folate deficiency; and (ix) presence of untreated thyroid disease.

The study was approved by the ethics committee of the Hospital Universitari Arnau de Vilanova-Santa Maria (Lleida, Spain) (CE-1218) and conducted according to the Declaration of Helsinki. The patient, the responsible caregiver, and the legal representative (when different from the responsible caregiver) signed an informed consent form.

### Study design

Consecutive patients arrived at the Cognitive Disorders Unit of the Hospital Universitari de Santa Maria (Lleida, Spain) and were assessed for their eligibility. A clinical evaluation was performed to investigate associated comorbidities and to collect sociodemographic and anthropometric data. Blood and CSF were obtained to respectively determine ApoE genotypes and the levels of AD pathological markers. Sleep-related evaluations included the application of the Epworth Sleepiness Scale (ESS) and an overnight PSG. Neuropsychological assessment was performed at baseline and after 12 months of follow-up.

### Clinical variables

The following variables were collected: sex, age, years of education, comorbidities (hypertension, diabetes mellitus, cardiopathy, periodic limb movements), and personal and family psychiatric and neurological history. Body mass index (BMI) was calculated as body weight (in kg)/height (in m^2^).

### Apolipoprotein E (ApoE) genotype

DNA was extracted from buffy coat cells using a Maxwell® RCS blood DNA kit (Promega, USA). Twenty microliters of DNA were used for ApoE genotyping by polymerase chain reaction (PCR). ApoE genotype was dichotomized as ApoE-ε4 homozygous or heterozygous carrier (ApoEε4 +) or not (ApoEε4 −).

### CSF biomarkers

The CSF samples were collected at baseline between 8:00 and 10:00 a.m. They were placed in polypropylene tubes, centrifuged at 2000 × g for 10 min at 4 °C, immediately frozen, and stored within 4 h in a − 80 °C freezer. The measurement of amyloid-beta protein (Aß42), T-tau, and P-tau was performed using commercial kits (Innotest® β-Amyloid-42; Innotest® hTAU Ag; and Innotest® Phospho-TAU181P, Fujirebio-Europe, Gent, Belgium). Neurofilament light (NF-L) was measured by commercial ELISA kit (Quidel, San Diego, CA). All determinations were performed in duplicate and in one round of experiments using one batch of reagents by board-certified laboratory technicians who were blinded to the clinical data. The intra-assay coefficients of variation were lower than 10% for internal quality control samples (two per plate). Based on previous data obtained by the research group, Aβ42 levels < 600 pg/ml were considered pathological [22].

### Epworth Sleepiness Scale (ESS)

Excessive daytime somnolence was assessed by the ESS. This questionnaire is composed of eight questions to assess the chance of falling asleep during different daily situations. Each question is rated on a 3-point scale, in which 0 represents no chance of occurrence, and 3 indicates a high chance of occurrence. The overall score ranges from 0 to 24 points. Higher scores represent increased daytime somnolence [23–25].

### Polysomnography (PSG)

The overnight PSG (Philips Respironics Alice 6 LDx, Philips, Murrysville, USA) was performed to assess the following variables: time spent in bed (in hours), total sleep time (in hours), sleep efficiency (in %, defined as the ratio between total sleep time and the time spent in bed), latency to N1 (in minutes, defined as the time spent awake until the first sleep episode), latency to REM sleep (in minutes, defined as the time spent asleep until the first REM sleep episode), the time spent in N1 stage (in %, defined as the percentage of time spent in N1 while asleep), the time spent in N2 stage (in %, defined as the percentage of time spent in N2 while asleep), the time spent in slow-wave sleep (SWS, also known as N3) (in %, defined as the percentage of time spent in SWS while asleep), the time spent in REM sleep (in %, defined as the percentage of time spent in REM sleep while asleep), the arousals index (defined as the mean number of awakening events per hour after the sleep onset), and the AHI (defined as the mean number of apnea and hypopnea events per hour during the time spent asleep).

The sleep staging and classification of the breathing cessation events that compose the AHI were performed according to the American Academy of Sleep Medicine (AASM) manual [26] by an experienced sleep technician who was blinded to this study. In this study, hypopneas were defined as the reduction of airflow that lasted more than 10 s leading to arousal or oxygen desaturation (represented by a decrease in the oxygen saturation greater than 3%).

The indexes of each one of the breathing cessation events that compose the AHI such as obstructive apneas, central apneas, mixed apneas, and hypopneas were calculated in three different ways: (i) considering the total sleep time ([number of the event of interest × 60]/total sleep time), (ii) considering only NREM sleep ([number of the event of interest during NREM sleep × 60]/time spent in NREM sleep), and (iii) considering only REM sleep ([number of the event of interest during REM sleep × 60]/time spent in REM sleep).

### Neuropsychological assessment

Patients underwent a neuropsychological evaluation through the MMSE at the beginning of the study and after 12 months of follow-up. The MMSE includes questions to evaluate different domains, such as attention, time and place orientation, and word recall. The scores of this test range from 0 to 30, and a higher score indicates better cognitive function [27, 28].

### Statistical analysis

Descriptive statistics were performed to report sociodemographic, clinical, and AD- and sleep-related data. Absolute and relative frequencies were used for qualitative data. The means (standard deviation (SD)) and medians (25th percentile; 75th percentile [p25;p75]) were estimated for quantitative variables with normal and nonnormal distributions, respectively. The normality of the distribution was assessed by the Shapiro–Wilk test.

The associations between the breathing cessation events that compose the AHI, sleep structure, and AD pathological markers were evaluated through linear regression models adjusted by age, sex, and BMI. Given the high collinearity among the breathing cessation events, we performed additional methods of data integration using partial least squares (PLS) regression to identify the pattern, in terms of AHI events, that better explained the relationship among the variables related to each outcome (sleep structure and AD pathological markers).

The associations between the breathing cessation events that compose the AHI and the cognitive decline at 12-month follow-up were evaluated through linear regression models adjusted by age, sex, BMI, years of education, and MMSE at baseline. Additionally, we performed multivariate analyses using PLS regression to identify the pattern, in terms of AHI events, most associated with the cognitive decline. The association between the first component of the PLS regression and cognitive decline was evaluated with Generalized Additive Model (GAM) adjusted for age, sex, BMI, years of education, and MMSE at baseline.

The *p*-value threshold defining statistical significance was set at < 0.05. Data management and statistical analyses were performed using R (version 4.0.1).

## Results

### Baseline characteristics

The population was composed of 116 patients diagnosed with mild-moderate AD (Table 1). Accordingly, the median [p_25_;p_75_] score of the MMSE at baseline was 23.0 [22.0;25.0] points. With 55.2% of females, the cohort presented a median age of 76.0 [72.0;80.0] years and a BMI of 27.7 [25.3;30.2] kg·m^−2^. Hypertension, periodic limb movements, and depression were the most frequent comorbidities with a prevalence of 62.9%, 44.7%, and 25.9%, respectively. Furthermore, the median AHI was 25.9 [15.1;48.5], with a sleep efficiency of 68.0% [53.7;78.2], and no daytime sleepiness according to the ESS (5.00 [3.00;8.00]).

**Table 1:** Baseline characteristics of the cohort

	**Global**
	*n* = 116
	*n (%), mean (SD) or median [p*_*25*_*;p*_*75*_*]*
***Sociodemographic data***	
Sex, female	64 (55.2%)
Age, years	76.0 [72.0;80.0]
BMI, kg·m^−2^	27.7 [25.3;30.2]
Education, years	7.0 [7.0;7.0]
***Comorbidities***	
Hypertension	73 (62.9%)
Diabetes mellitus	23 (19.8%)
Cardiopathy	25 (21.6%)
Depression	30 (25.9%)
Periodic limb movements	46 (44.7%)
***Alzheimer’s disease parameters***	
*Cognitive function*	
MMSE	23.0 [22.0;25.0]
*Biomarkers levels*	
Aβ42, pg/ml	527 [415;643]
T-tau, pg/ml	464 [324;618]
P-tau, pg/ml	73.4 [52.3;94.4]
*Genetic risk*	
ApoEε4 +	60 (53.1%)
***Sleep parameters***	
*Questionnaire*	
Epworth sleepiness scale	5.00 [3.00;8.00]
*Polysomnography*	
Total sleep time, hours	4.57 [3.51;5.38]
Sleep efficiency, %	68.0 [53.7;78.2]
N1, %	12.1 [8.14;18.4]
N2, %	24.3 (11.3)
SWS, %	17.2 [8.27;24.5]
REM sleep, %	6.74 [2.48;11.2]
Latency to N1, minutes	21.0 [9.26;41.8]
Latency to REM, minutes	161 [99.6;250]
AHI	25.9 [15.1;48.5]

*AHI* apnea–hypopnea index, *ApoEε4* + apolipoprotein E carrier, *Aβ42*, amyloid-beta protein, *BMI* body mass index, *MMSE* Mini-Mental State Examination, *n* number, *p* percentile, *P-tau* phosphorylated-tau, *SD* standard deviation, *SWS* slow-wave sleep, *T-tau* total-tau. Missings: biomarkers, 12; ApoEε4 + , 3

### Frequency of the breathing cessation events

**Fig. 1:**
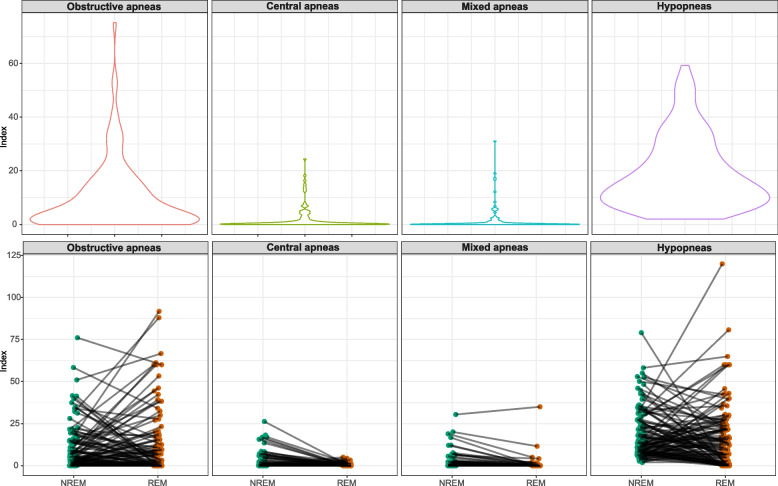
Frequency of the breathing cessation events composing the AHI among mild-moderate Alzheimer’s disease patients. AHI, apnea–hypopnea index

The breathing cessation events composing the AHI were evaluated to determine their frequency considering the total sleep time and each sleep stage individually. Hypopneas were the most frequent event considering the total sleep time with a median index of 14.7 [8.32;25.8], followed by the obstructive apneas (median index of 5.00 [0.86;14.1]) (Table S1). Such scenario was maintained considering each sleep stage individually, with central and mixed apneas presenting a modest frequency. Furthermore, comparisons between NREM and REM sleep demonstrated that the distribution of hypopneas and obstructive apneas among these sleep stages varied significantly according to each patient (Fig. 1).

### Breathing cessation events and the sleep structure

Linear regression models adjusted by age, sex, and BMI revealed distinct patterns of associations between the breathing cessation events composing the AHI and the sleep structure (Table 2). The index of obstructive apneas presented positive associations with the arousals index (effect size [95% CI] of 0.78 [0.651 to 0.909]) and the percentage of time in N1 (0.334 [0.165 to 0.503]) in detriment of the time spent in SWS (− 0.227 [− 0.405 to − 0.049]). Higher central and mixed apneas indexes were related to an increased percentage of time in N1 (central: 0.289 [0.120 to 0.457]; mixed: 0.199 [0.024 to 0.373]), but no compensatory association with a decrease in the percentage of time in SWS was observed. Differently, the hypopnea index was only associated with the arousals index (0.521 [0.356 to 0.685]), and no alterations in relation to the sleep stages were detected.

**Table 2:** Sleep structure according to the breathing cessation events

	*Effect size (95% CI)*	*p-value*
**Obstructive apnea index**		
N1	0.334 (0.165 to 0.503)	< 0.001
N2	0.104 (− 0.084 to 0.292)	0.281
SWS	− 0.227 (− 0.405 to − 0.049)	0.014
REM sleep	− 0.059 (− 0.251 to 0.133)	0.549
Arousals	0.78 (0.651 to 0.909)	< 0.001
**Central apnea index**		
N1	0.289 (0.120 to 0.457)	0.001
N2	− 0.151 (− 0.337 to 0.035)	0.113
SWS	− 0.082 (− 0.262 to 0.098)	0.377
REM sleep	− 0.063 (− 0.253 to 0.127)	0.518
Arousals	0.173 (− 0.013 to 0.359)	0.071
**Mixed apnea index**		
N1	0.199 (0.024 to 0.373)	0.026
N2	0.145 (− 0.041 to 0.331)	0.128
SWS	− 0.053 (− 0.233 to 0.127)	0.565
REM sleep	0.071 (− 0.119 to 0.261)	0.464
Arousals	0.425 (0.254 to 0.595)	< 0.001
**Hypopnea index**		
N1	0.029 (− 0.149 to 0.207)	0.747
N2	0.135 (− 0.051 to 0.321)	0.158
SWS	− 0.123 (− 0.305 to 0.059)	0.188
REM sleep	− 0.094 (− 0.286 to 0.098)	0.335
Arousals	0.521 (0.356 to 0.685)	< 0.001

Linear regression models adjusted by age, sex, and BMI representing the sleep structure according to the breathing cessation events that compose the AHI

*AHI* apnea–hypopnea index, *BMI* body mass index, *CI* confidence interval, *SWS* slow-wave sleep

Additional analyses were performed to investigate whether the pattern of associations between the components of the AHI and the sleep structure would be distinct considering the events occurring in each sleep stage (NREM or REM sleep) separately (Table S2). Contemplating the events occurring during NREM sleep only, there was a similar pattern of associations compared to those previously observed. Nevertheless, such pattern was not detected in the analysis considering exclusively the events occurring during REM sleep.

To counteract the high collinearity among the breathing cessation events and among the sleep stages, we performed methods of data integration based on PLS regression. The findings corroborated those previously observed, revealing that the most relevant pattern among the sample was mainly characterized by the presence of obstructive apneas and an increased number of arousals (Fig. S1).

### Breathing cessation events and the pathological markers of Alzheimer’s disease

Linear regression models adjusted by age, sex, and BMI revealed that a higher hypopnea index was associated with an increased P-tau/Aβ42 ratio with an effect size of 0.217 (0.017 to 0.417) (Table 3). In addition, higher indexes of obstructive and mixed apneas were related to increased levels of NF-L (obstructive: 0.252 [0.013 to 0.491]; mixed: 0.229 [0.043 to 0.415]). A similar pattern of associations between the breathing cessation events and the pathological markers of AD was observed when contemplating the events occurring during NREM sleep only (Table S3). Nevertheless, no associations were detected in the analysis considering exclusively the events occurring during REM sleep.

**Table 3:** Pathological markers of Alzheimer’s disease according to the breathing cessation events

	*Effect size (95% CI)*	*p-value*
**Obstructive apnea index**		
Aβ42	0.108 (− 0.086 to 0.302)	0.276
T-tau	0.035 (− 0.161 to 0.231)	0.723
P-tau	0.011 (− 0.185 to 0.207)	0.915
NF-L	0.252 (0.013 to 0.491)	0.042
P-tau/Aβ42	− 0.028 (− 0.226 to 0.17)	0.782
**Central apnea index**		
Aβ42	0.022 (− 0.19 to 0.234)	0.84
T-tau	0.148 (− 0.064 to 0.36)	0.172
P-tau	0.052 (− 0.162 to 0.266)	0.634
NF-L	0.09 (− 0.126 to 0.306)	0.414
P-tau/Aβ42	0.023 (− 0.193 to 0.239)	0.832
**Mixed apnea index**		
Aβ42	0.019 (− 0.171 to 0.209)	0.848
T-tau	− 0.017 (− 0.207 to 0.173)	0.859
P-tau	0.027 (− 0.163 to 0.217)	0.785
NF-L	0.229 (0.043 to 0.415)	0.019
P-tau/Aβ42	0.013 (− 0.179 to 0.205)	0.898
**Hypopnea index**		
Aβ42	− 0.109 (− 0.309 to 0.091)	0.289
T-tau	− 0.033 (− 0.235 to 0.169)	0.748
P-tau	0.2 (0.002 to 0.398)	0.051
NF-L	− 0.08 (− 0.310 to 0.150)	0.49
P-tau/Aβ42	0.217 (0.017 to 0.417)	0.035

Linear regression models adjusted by age, sex, and BMI representing the pathological markers of AD according to the breathing cessation events that compose the AHI

*AD* Alzheimer’s disease, *AHI* apnea–hypopnea index, *Aβ42* amyloid-beta protein, *BMI* body mass index, *CI* confidence interval, *NF-L* neurofilament light, *P-tau* phosphorylated-tau, *T-tau* total-tau

The PLS regression analysis to counteract the high collinearity among the breathing cessation events and among the pathological markers of AD revealed that the component with the most relevance was characterized by the number of obstructive apneas and hypopneas mostly, without a clear distinctiveness among the AD pathological markers (Fig. S2).

### Breathing cessation events and the cognitive decline

**Fig. 2:**
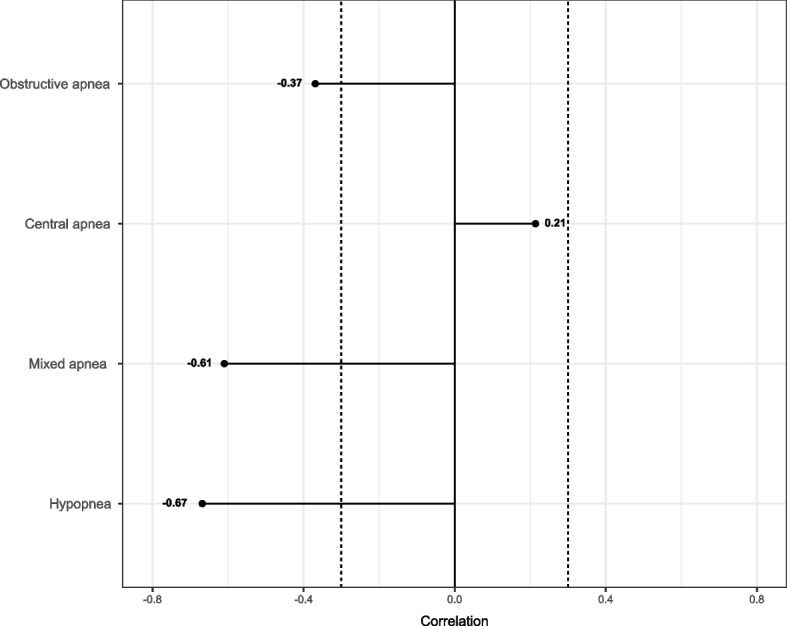
PLS regression analysis (cognitive decline). The findings reveal that the pattern of breathing cessation events that better explains the variability of the sample in terms of cognitive decline is mainly characterized by the presence of hypopneas. PLS, partial least squares

**Fig. 3:**
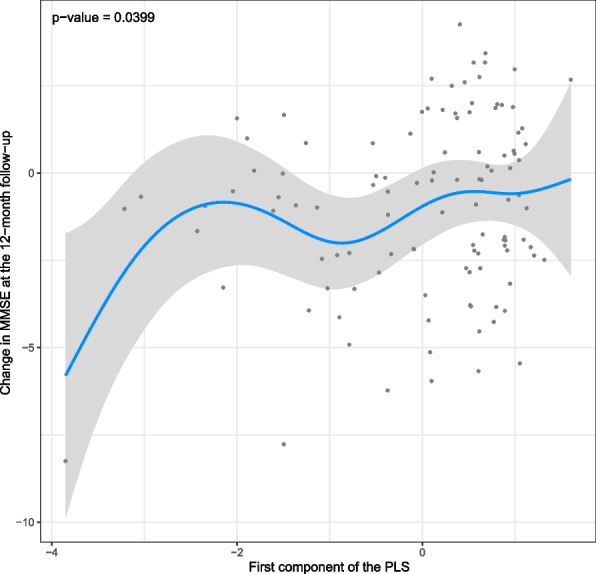
Cognitive decline according to the first component of the PLS regression analysis. Lower values of the first component of the PLS (*X*-axis) represent a higher number of hypopneas. Lower values of change in MMSE at the 12-month follow-up (*Y*-axis) represent a greater decrease in MMSE at the 12-month follow-up (i.e., higher cognitive decline). MMSE, Mini-Mental State Examination; PLS, partial least squares

Linear regression models adjusted by age, sex, BMI, years of education, and MMSE at baseline did not reveal substantial associations between the breathing cessation events composing the AHI and the cognitive decline (Table S4). We performed an additional analysis to evaluate whether distinct patterns in terms of AHI events would be related to a differential cognitive decline. The results revealed that the pattern that better explained the variability of the sample in terms of cognitive decline was characterized by the presence of hypopneas, possibly associated with the presence of mixed and obstructive apneas and inversely related to the presence of central apneas (Fig. 2). Accordingly, such pattern was associated with an increased cognitive decline at the 12-month follow-up (0.509 [0.025 to 0.992]) (Fig. 3).

Given that the previous analyses suggested a negligible influence of the events occurring during REM sleep, we performed an additional PLS regression considering only the events occurring during NREM sleep. Similar findings were obtained, with the pattern that better explained the variability of the sample in terms of cognitive decline being characterized by the presence of hypopneas (Fig. S3). Furthermore, such pattern was associated with a higher cognitive decline after 12 months of follow-up, with an effect size of 0.476 (− 0.001 to 0.952) after adjusting for age, sex, BMI, years of education, and MMSE at baseline.

## Discussion

In the current study, we first characterized the breathing cessation events composing the AHI among our cohort of mild-moderate AD patients. Such analysis revealed a high frequency of hypopneas followed by the presence of obstructive apneas. The distribution of the events along the sleep stages presented substantial variability among the patients. Furthermore, each event presented a distinct pattern of associations with sleep. Apneas were associated with arousals and sleep lightening whereas hypopneas, despite being related to an increased number of arousals, did not affect the sleep structure. Nevertheless, hypopneas were associated with the levels of the classical markers of AD. More importantly, the hypopnea index was the most relevant factor to predict an increased cognitive decline at the 12-month follow-up.

The inclusion of apneas and hypopneas in the same metric to diagnose and establish the severity of the so-called OSA generates an implicit assumption that these events are similar in relation to their clinical impact. While some studies corroborate this line [29], others challenge the absence of distinctiveness among them [30, 31]. Kulkas and collaborators (2017) demonstrated that obstructive apneas are associated with more severe SpO2 desaturation compared to hypopneas, suggesting a higher relevance of the first when estimating the severity of OSA and associated long-term cardiovascular outcomes [30]. Several studies also reveal distinct outcomes according to the used criteria for the classification of hypopneas, highlighting the relevance of a detailed analysis of the events composing the AHI [31–36]. To our knowledge, this is the first study to address this matter, demonstrating the distinctiveness between hypopneas and obstructive apneas in terms of associated clinical outcomes among AD patients.

We observed that obstructive apneas were related to an increase in the time spent in N1 in detriment of the time spent in SWS, besides an association with an increased number of arousals. Also, these events were related to increased levels of NF-L, a marker for the axonal damage and cognitive decline of patients with AD [37–39]. Accordingly, we have previously demonstrated that patients with a propensity to spend most of the time in the lighter sleep stage (light sleepers) present an increased probability of having high NF-L levels compared with those individuals with a propensity to deepen their sleep (deep sleepers) [40]. Differently, despite the association with an increased number of arousals, the hypopneas were not related to alterations in the sleep structure. Nevertheless, the hypopnea index was associated with the levels of classical markers of AD and was the most relevant breathing cessation event to predict an increased cognitive decline at the 12-month follow-up. This suggests a possible contribution of arousals within this context regardless of whether alterations in the sleep structure are present or detected.

Based on the guidelines of the American Academy of Sleep Medicine (AASM), hypopneas and obstructive apneas are differentiated by the degree of airflow decrease (30% to 89% versus ≥ 90%, respectively) [26]. In consequence, obstructive apneas are associated with higher oxygen desaturation, which suggests a greater clinical impact in comparison to hypopneas [30]. Similarly, our findings revealed a stronger association between obstructive apneas and arousals than that involving hypopneas, which reinforces the greater risk of clinical consequences related to the apneic events. Still, our data indicate a distinguished role of hypopneas on the cognitive decline after 12 months of follow-up. A possible explanation for such outcome could be related to the 3-times higher frequency of hypopneas among our population compared to that of obstructive apneas. Future studies will be necessary to evaluate such interesting finding, confirming whether there are relationships of causality in this regard. Also, larger cohorts will provide the required variability to investigate differences between patients who have a pattern of breathing cessation events mostly composed of hypopneas and those in whom obstructive apneas are the most prevalent.

## Limitations

The current findings should be interpreted in light of some aspects: (i) due to the sample size and the exploratory nature of this study, our data were not adjusted for multiple comparisons; (ii) given previous demonstrations of a differential effect of OSA on cognitive function depending on the cognitive status of the subjects and/or presence of AD pathology [8, 12, 17, 18], the associations herein observed cannot be extrapolated to cognitively healthy subjects or cognitively unimpaired patients with AD pathology; (iii) the sleep evaluation revealed a low percentage of time spent in REM sleep, which might have prevented the observation of associations in this regard; (iv) the discreet prevalence of central and mixed apneas in addition to the high collinearity among the components of the AHI may have led to associations influenced by the most prevalent events such as hypopneas and obstructive apneas; (v) although the objective of this study was limited to investigate whether the AHI components associated with increased sleep disruption would be the ones linked to worse outcomes, the influence of the respiratory burden related to each event should not be ruled out; (vi) given the observational design of this study, it is not possible to confirm causality and directionality between the associations of interest.

## Conclusions

In summary, we demonstrated that the hypopneas and the obstructive apneas are the components that define the AHI among consecutive patients with mild-moderate AD. These events were distinctively associated with the sleep structure, the levels of pathological markers of AD, and cognitive decline, suggesting that the OSA does have an influence once patients are diagnosed with symptomatic disease up to a certain extent. This altogether highlights the importance of a patient-centered approach, with a comprehensive and detailed analysis of the AHI to effectively predict the different outcomes and tailor the appropriate therapeutic strategies.

## Supplementary Information

**Additional file 1: Table S1. **Frequency of the breathing cessation events.

**Additional file 2: Table S2.** Sleep structure according to the breathing cessation events in each sleep stage.

**Additional file 3: Table S3.** Pathological markers of Alzheimer’s disease according to the breathing cessation events in each sleep stage.

**Additional file 4: Table S4.** Cognitive decline according to breathing cessation events.

**Additional file 5: Figure S1.** PLS regression analysis (sleep structure). The findings reveal that the most relevant pattern in relation to the breathing cessation events and sleep structure is mainly characterized by the presence of obstructive apneas and an increased number of arousals. PLS, partial least squares; SWS, slow wave sleep.

**Additional file 6: Figure S2.** PLS regression analysis (pathological markers of Alzheimer’s disease). The findings reveal that the most relevant pattern in relation to the breathing cessation events and pathological markers of AD is characterized by the number of obstructive apneas and hypopneas mostly, without a clear distinctiveness among the Alzheimer’s disease pathological markers. AD, Alzheimer’s disease; Aβ42, amyloid-beta protein; NF-L, neurofilament light; P-tau, phosphorylated-tau; PLS, partial least squares; T-tau, total-tau.

**Additional file 7: Figure S3.** PLS regression analysis (cognitive decline). The findings reveal that the pattern of breathing cessation events occurring during NREM sleep that better explains the variability of the sample in terms of cognitive decline is mainly characterized by the presence of hypopneas. PLS, partial least squares.

## Data Availability

The datasets used and/or analyzed during the current study are available from the corresponding author on reasonable request.

## Abbreviations

AD: Alzheimer’s disease
AHI: Apnea-hypopnea index
ApoE: Apolipoprotein E
Aβ42: Amyloid-beta protein
BMI: Body mass index
CSF: Cerebrospinal fluid
ESS: Epworth sleepiness scale
MMSE: Mini-Mental State Examination
NF-L: Neurofilament light
OSA: Obstructive sleep apnea
P-tau: Phosphorylated-tau
PSG: Polysomnography
SWS: Slow-wave sleep
T-tau: Total-tau
