# Supplementary figures and images for: Breathing cessation events that compose the apnea–hypopnea index are distinctively associated with the adverse outcomes in Alzheimer’s disease
Source: Alzheimers Res Ther. 2023 Jul 14;15:123. doi: 10.1186/s13195-023-01266-x (PMC10347810; doi:10.1186/s13195-023-01266-x)

A

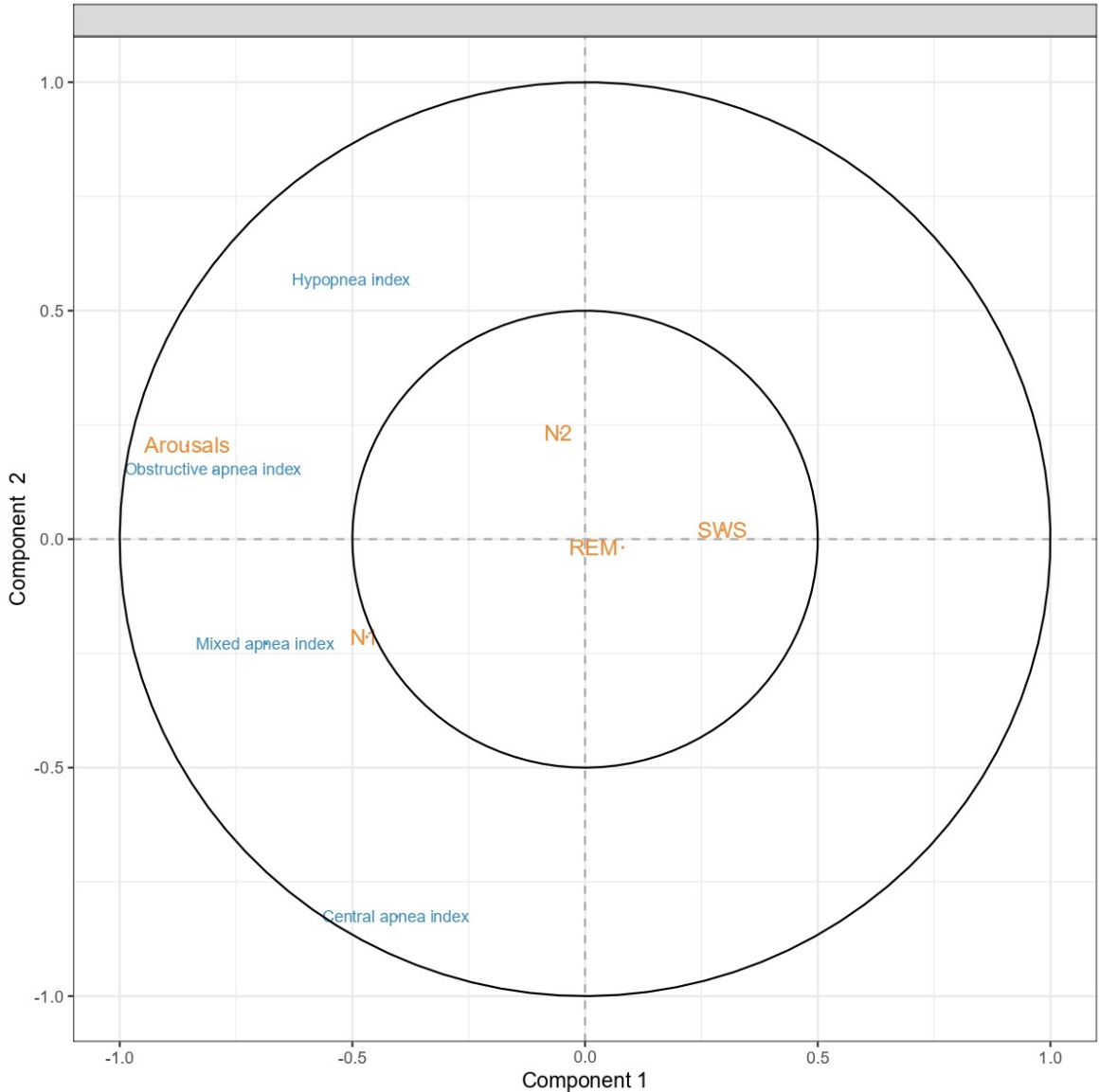

B

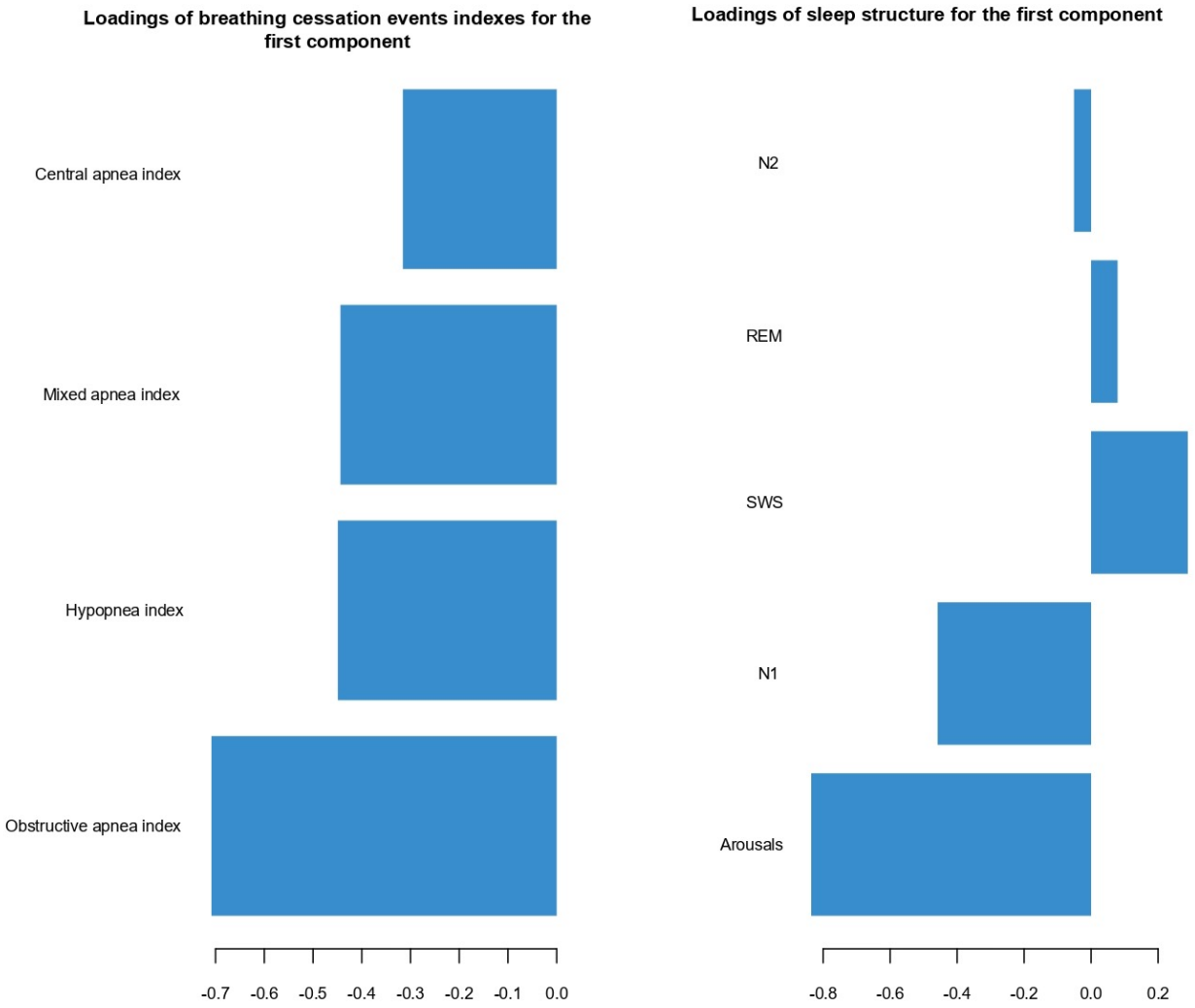

Supplement: Supplementary file 5 — Additional file 5: Figure S1. PLS regression analysis (sleep structure). The findings reveal that the most relevant pattern in relation to the breathing cessation events and sleep structure is mainly characterized by the presence of obstructive apneas and an increased number of arousals. PLS, partial least squares; SWS, slow wave sleep. [file 13195_2023_1266_MOESM5_ESM.pdf]

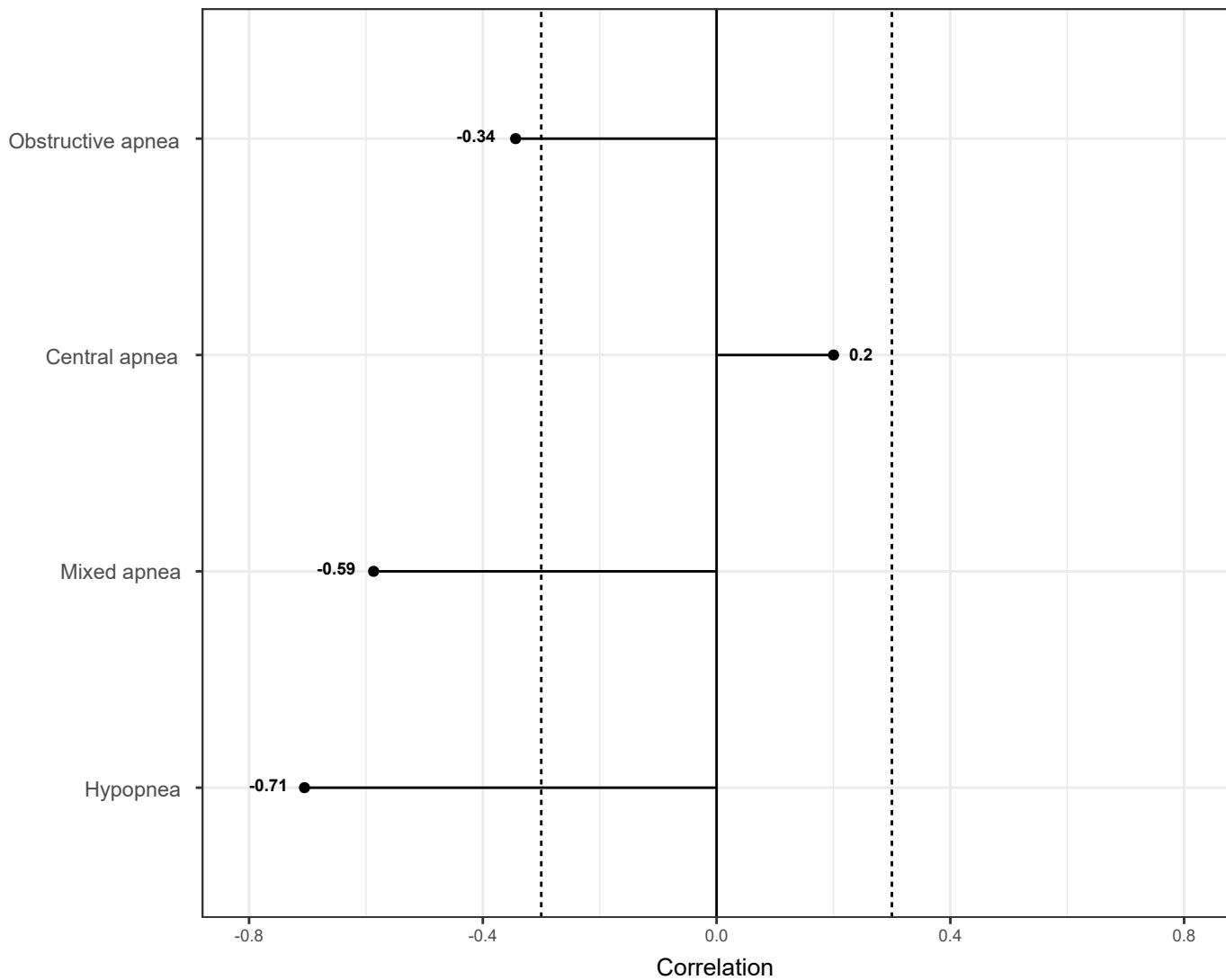

Supplement: Supplementary file 7 — Additional file 7: Figure S3. PLS regression analysis (cognitive decline). The findings reveal that the pattern of breathing cessation events occurring during NREM sleep that better explains the variability of the sample in terms of cognitive decline is mainly characterized by the presence of hypopneas. PLS, partial least squares. [file 13195_2023_1266_MOESM7_ESM.pdf]
