# Supplementary material for: Breathing cessation events that compose the apnea–hypopnea index are distinctively associated with the adverse outcomes in Alzheimer’s disease
Source: Alzheimers Res Ther. 2023 Jul 14;15:123. doi: 10.1186/s13195-023-01266-x (PMC10347810; doi:10.1186/s13195-023-01266-x)
Supplement: Supplementary file 6 — Additional file 6: Figure S2. PLS regression analysis (pathological markers of Alzheimer’s disease). The findings reveal that the most relevant pattern in relation to the breathing cessation events and pathological markers of AD is characterized by the number of obstructive apneas and hypopneas mostly, without a clear distinctiveness among the Alzheimer’s disease pathological markers. AD, Alzheimer’s disease; Aβ42, amyloid-beta protein; NF-L, neurofilament light; P-tau, phosphorylated-tau; PLS, partial least squares; T-tau, total-tau. [file 13195_2023_1266_MOESM6_ESM.pdf]

A

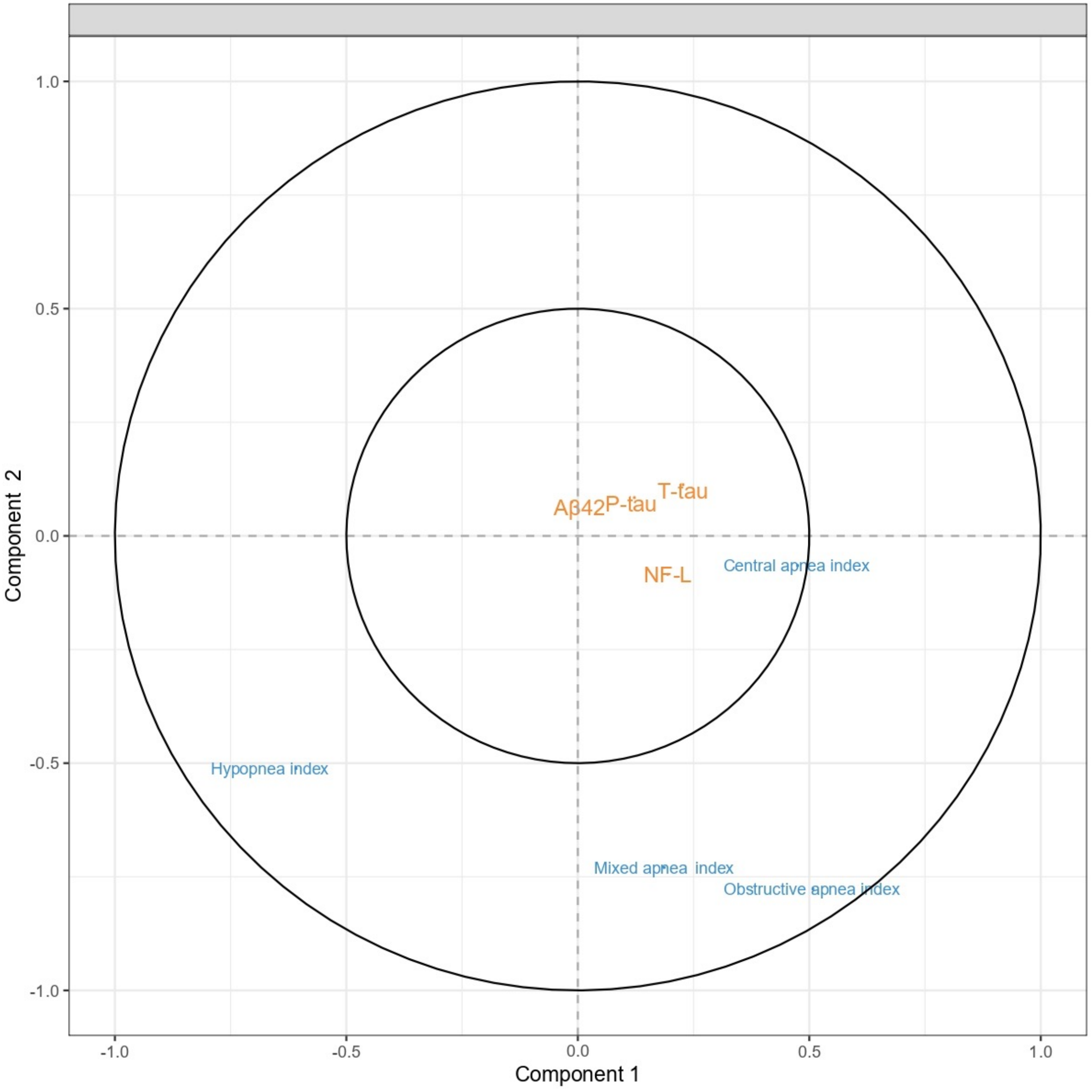

1.0
0.5
0.0
-0.5
-1.0
Component 2
Aβ42
P-tau
T-tau
NF-L
Central apnea index
Hypopnea index
Mixed apnea index
Obstructive apnea index
-1.0
-0.5
0.0
0.5
1.0
Component 1

B

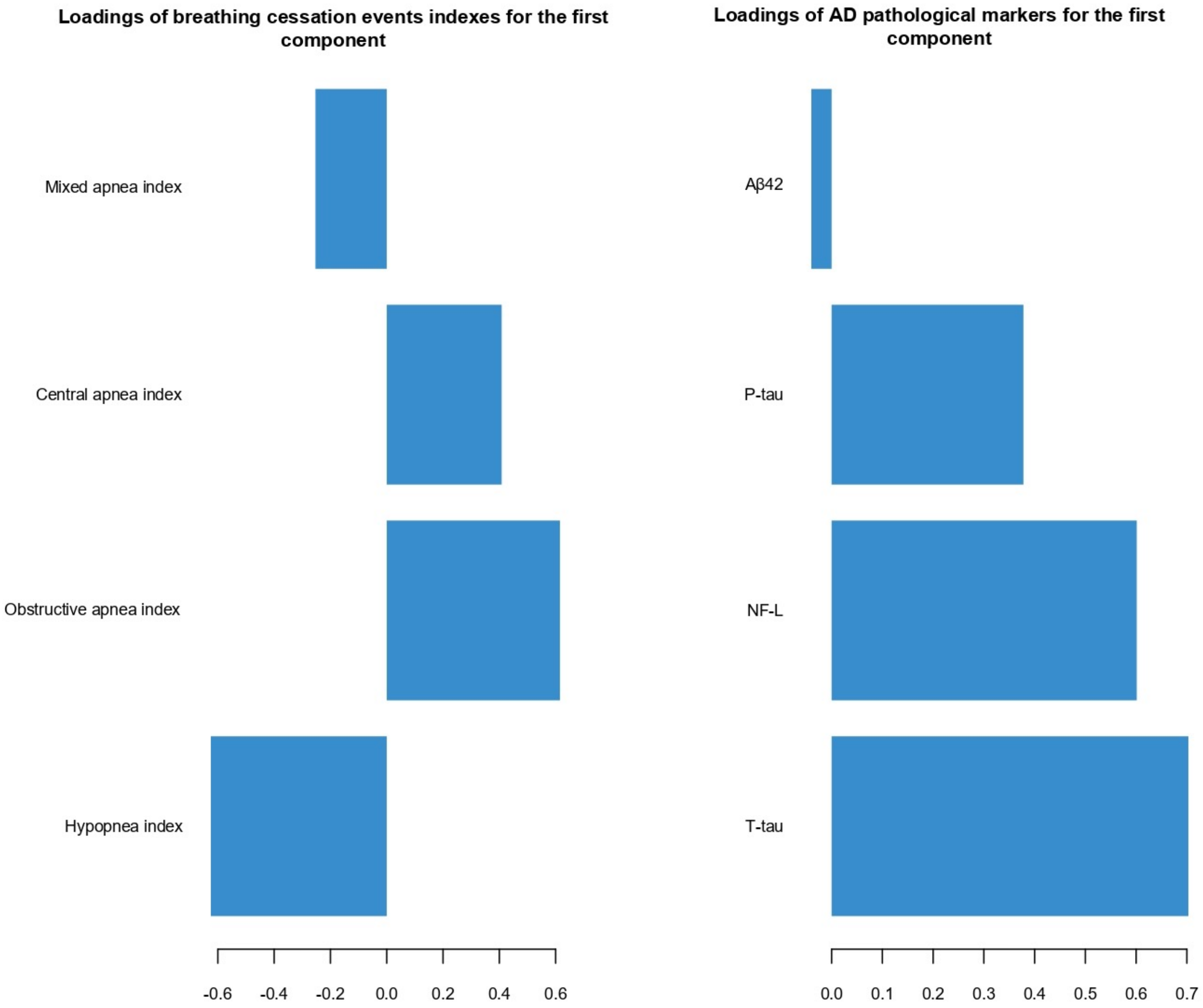

Loadings of breathing cessation events indexes for the first component
Mixed apnea index
Central apnea index
Obstructive apnea index
Hypopnea index
-0.6
-0.4
-0.2
0.0
0.2
0.4
0.6
Loadings of AD pathological markers for the first component
Aβ42
P-tau
NF-L
T-tau
0.0
0.1
0.2
0.3
0.4
0.5
0.6
0.7
